# A rule-based algorithm for automatic bond type perception

**DOI:** 10.1186/1758-2946-4-26

**Published:** 2012-10-31

**Authors:** Qian Zhang, Wei Zhang, Youyong Li, Junmei Wang, Liling Zhang, Tingjun Hou

**Affiliations:** 1 Institute of Functional Nano & Soft Materials (FUNSOM) and Jiangsu Key Laboratory for Carbon-Based Functional Materials & Devices, Soochow University, Suzhou, Jiangsu 215123, China; 2 College of Pharmaceutical Sciences, Soochow University, Suzhou, Jiangsu 215123, China; 3 Department of Biochemistry, The University of Texas Southwestern Medical Center, 5323 Harry Hines Blvd, Dallas, TX, 75390, USA

**Keywords:** Bond type perception, Bond order, Chemical bond, Molecular modeling

## Abstract

Assigning bond orders is a necessary and essential step for characterizing a chemical structure correctly in force field based simulations. Several methods have been developed to do this. They all have advantages but with limitations too. Here, an automatic algorithm for assigning chemical connectivity and bond order regardless of hydrogen for organic molecules is provided, and only three dimensional coordinates and element identities are needed for our algorithm. The algorithm uses hard rules, length rules and conjugation rules to fix the structures. The hard rules determine bond orders based on the basic chemical rules; the length rules determine bond order by the length between two atoms based on a set of predefined values for different bond types; the conjugation rules determine bond orders by using the length information derived from the previous rule, the bond angles and some small structural patterns. The algorithm is extensively evaluated in three datasets, and achieves good accuracy of predictions for all the datasets. Finally, the limitation and future improvement of the algorithm are discussed.

## Background

The advances in protein cloning, expression, labeling, purification, protein crystallization and structure determination has resulted in a rapid increase in protein structures having been determined [1]. Nearly 45 years ago, the process to determine a small-molecule crystal structure consumed several months, and the bottlenecks were the data-collection, structure-solution and structure-refinements stages [2]. In recent years, X-ray crystallography and solution NMR, the two major techniques broadly used for determining the atomic structures of biomolecules, have been improved substantially [3]. There already have 85212 PDB files (2012.10.09) deposited in the RCSB Protein Data Bank [4]. For proteins and nucleic acids, bond orders can be easily deduced but it is not so easy for other types of molecules, such as ligands [5]. Because the resolutions of a lot of X-ray structures are not high enough, the hydrogen atoms and bond type information usually cannot be identified and even the coordinates have large errors, which bring difficulty in confirming the bond types of the structures. Although we can manually assign the bond types for the co-crystalized ligands, the procedure is tedious and time-consuming if one wants to deal with a large number of structures.

Many methods have been developed for automatically detecting the chemical structures [5-14]. For example, Baber et al. developed an algorithm to assign the bond orders by comparing the bond length with ideal values, and this algorithm uses a confidence value for each bond to solve the conflicts between coordination state and bond order [6]. Hendlich and co-workers proposed the BALI method to automatically assign bonds for protein ligand in the Brookhaven Protein Data Bank, and the assignment procedure of BALI has several stages, including the recognition of simple functional groups, the perception of ring systems, and the optimization of the assignment of alternating single and double bonds to networks of sp^2^ hybridized atoms [9]. Froeyen and Herdewijn proposed a procedure to assign double and triple bonds in small molecules from simple PDB files; the method uses sigma bonds connectivity and atom symbols as the input, and then assigns double and triple bonds by translating the octet equations into an integer liner program [8]. Bruno et al. designed an algorithm to assign bonds for the molecules in the CSD (Cambridge Structural Database). Bruno’s algorithm divides the whole molecule into small fragments at first, then calculates the frequencies of the fragments in the CSD and uses geometry tests to obtain conditional probability, and finally apply Bayes’ formula to assign structures [7]. Zhao’s method detects the hybridization states of atoms and then uses functional groups and length rules to determine the bond types [13]. Wang and Case applied a recursive algorithm to assign bond types using the atom symbols and bond connectivity table as input. In Wang’s algorithm, the best assignment is recognized when the total bond order of each atom satisfying the predefined atomic valence parameter [14].

All these available methods have their own advantages, but at the same time, have some disadvantages too. For example, Labute used Maximum Weighted Matching algorithm to assign bond orders instead of using group patterns in order to avoid mis-constructing structures that are similar to group patterns [10]. However, due to this advantage, the structures with large coordinate errors usually cannot be fixed because this method heavily relies on precise coordinates. Opposite to Labute’s work, the algorithm developed by Zhao et al. assigns the hybridization states for each atom first, and then uses a lot of functional groups to assign bond orders [13]. Using functional groups can avoid the disadvantage of poor coordinates, but the false detections of hybridization and similar structures will impair bond perception. Wang and Case’s algorithm, which has been implemented in the Antechamber module of AMBER package, is robust and widely used among the AMBER community; however, their method fails if the input molecule has open valences.

**Figure 1:**
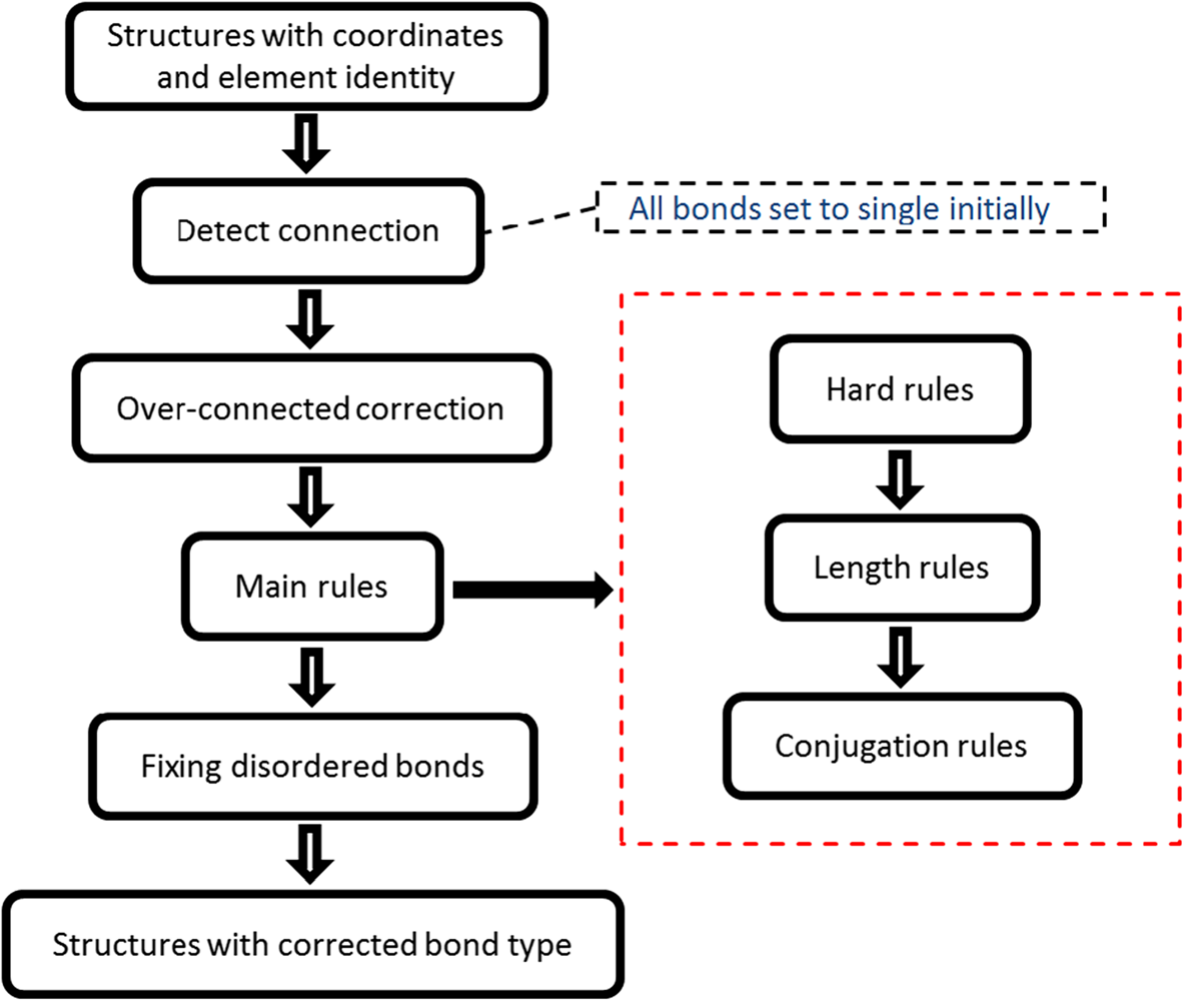
The flow chart of the algorithm.

In our work, in order to overcome the disadvantages mentioned above, we designed a new algorithm for the bond perception based on three different sets of rules: hard rules, length rules and conjugation rules. Moreover, in this algorithm, we employed small structural patterns to assign the matched groups rather than using large ones, which can avoid the errors caused by similar structures. In conjugation rules, we divide the bonds that remain unfixed into several groups, and the bonds that connect to each other are in the same group. And then, in order to enhance the reliability of the results we fix these groups one by one instead of fixing bonds separately. The flow chart of our algorithm is shown in Figure 1. Compared with the other algorithms, our algorithm has the following advantages: (1). this algorithm has a better tolerance of coordinate error; (2). it can avoid many false perceptions because of the employment of fewer patterns; (3). it can select a better structure from isomers by using the bond entropy; (4). it can assign the bond orders for the structures without hydrogen.

## Results and discussion

We used three data sets to validate our algorithm. The first data set collected by Ricketts et al. [15] has 17 ligands extracted from the PDB files (PDB entries are 1cla, 1fcb, 1fx1, 1gox, 2aat, 2dhf, 2gbp, 2trm, 3cpp, 3ptb, 4dfr, 4xia, 5xia, 7dfr, 8atc, and 8rsa), and it has been extensively used for testing the algorithms of bond assignment. Therefore, this data set was also used by us to evaluate our algorithm.

**Figure 2:**
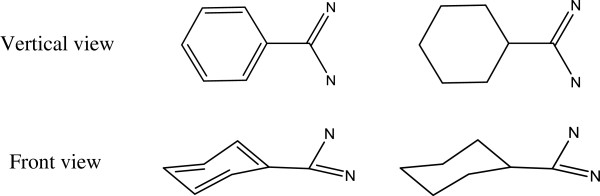
The representation of the ligand in 2trm in different orientations.

The performance of our algorithm and the other four algorithms for the first data set is summarized in Table 1. In all these algorithms, the algorithm developed by Sayle et al. correctly fixed all the structures. It should be noted that all the structures were used to train the Sayle’s algorithm [12], and therefore it is understandable that the Sayle’s algorithm can fix all the structures in the first data set. The Labute’s algorithm correctly perceived 14 structures, the Baber’s algorithm 7, and the Meng’s algorithm 5. Our algorithm correctly perceived 16 structures, and only failed on 1 structure in 2trm. The ligand in 2trm is benzamidine, and our method detects a cyclohexene ring instead of a benzene ring. From the front view shown in Figure 2, it can be found that the ring of six carbons is just like cyclohexane in its chair form, and therefore our algorithm assigns the six C-C bonds to single bonds because all these atoms are not on the same plane.

**Table 1:** The performance of different algorithms on the first data set

**Algorithm**	**Success number**	**Success rate**
Baber	7	41.2%
Meng	5	29.4%
Sayle	17	100%
Labute	14	82.4%
Our	16	94.1%

According to the evaluation results for the first data set, our algorithm has excellent performance among those algorithms. Then, a larger data set with 179 entries (hydrogen suppressed) from the PDB was used for the further assessment of our algorithm. Labute et al. [10] and Zhao et al. [13] used this data set to test their algorithms. The PDB codes of these structures in the second data set are listed in Additional file 1: Table S2. Among the 179 structures, 170 were correctly interpreted (95.0%) by our algorithm and 9 were incorrectly perceived. The Labute’s algorithm failed on 11 structures and the Zhao’s algorithm on 9 structures. Moreover, two popular molecular simulation packages, including Discovery Studio (version 2.5) [16] and SYBYL-X (version 1.3) [17], were used to perceive the molecules in the second data set. According to the prediction results, Discovery Studio failed on 25 structures and SYBYL even failed on 46 structures. That is to say, the performance of Discovery Studio and SYBYL is much worse than that of our algorithm. The problematic structures predicted by our algorithm are listed in Table 2:

1. 1cps and 3dfr: several bonds in 1cps and 3dfr that were supposed to be double are too long, and then they were assigned to single due to their lengths. The lengths of two C-C bonds in the ring of 1cps are 1.62Å and 1.57Å, respectively, and are larger than 1.49Å. The length of a C-C bond in 3DFR is 1.54Å and was set to single according to length rules.

2. 1bzm, 1nnb, 1rne and 3fx2: for the ligands extracted from these four PDB files, several double bonds were incorrectly detected because they are not on the plane. We calculated the smallest angle between the bond and plane, and found that none of them satisfied the plane rule, and therefore all of them were set to single bonds. Here we may find two solutions to improve our algorithm: design and apply more structural patterns or modify the criteria used to check the plane.

3. 5tln and 8xia: the terminal bond C-O is supposed to be double but the angle of O-C-C deviates greatly from 120° (90.56° and 97.63°, respectively), so our algorithm set their bond orders to 1 based on our rules. We suspect inadequate refinement of the ligand geometry because the same functional groups in the other entries can be correctly perceived by our algorithm.

4. 5tim: two sulfurs in 5tim are not connected to each other in the correct structure. But because of short distance, a connection between them was established. Moreover, due to the non-existence of hydrogen, and the maximum connection number of sulfur is 4, and therefore we could not find out the false connection by detecting the number of the connected atoms and the valence. One way that may solve this problem is to calculate the hybridization of sulfur. But because no hydrogen is provided, even if the two sulfurs are connected, we still cannot find the mistake just based on the structure.

**Table 2:** The nine failed structures predicted by our algorithm

**PDB code**	**Predicted structure**	**Correct structure**
1bzm		
1cps		
1nnb		
1rne		
3dfr		
3fx2		
5tim		
5tln		
8xia		

Compared with the results predicted by the Labute’s algorithm, we find that 2cgr, 3dfr and 5tln were not correctly detected by both of the Labute’s algorithm and our algorithm. Labute ascribed these errors to questionable or ambiguous geometry. According to our analysis, we think that the wrong perception of structures can be explained by the following two reasons: awkward structures and imperfect algorithm. For awkward coordinates, we may analyze the large prior data to get an amendment between PDB files and true structures instead of using patterns, because if more patterns are used, more errors may be introduced. But on the other hand, we still need some structural patterns to deal with the coordinate errors when the resolution of a PDB file is not high. Furthermore, the judgment of atom connection is also an important point to which we need to pay more attention.

**Figure 3:**
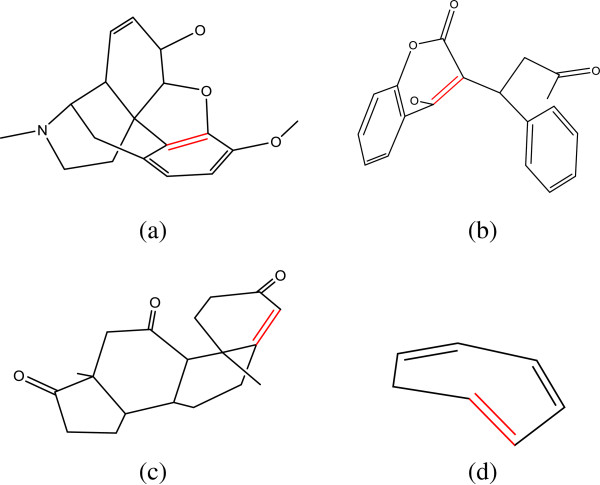
**Four failed small molecules with the bond types beyond the rules in our algorithm (the unfixed bonds are colored in red).**

Finally, we tested our algorithm on an extensive collection of 1290 small molecules (with hydrogen) used by us to train a prediction model of solubility [18]. For this dataset, our algorithm achieves the prediction accuracy is 97.8% (1265 structures were correctly perceived). Out of the 25 failed structures, most of them (19) were found to have unusual bond lengths. For those failed cases, two possibilities lead to false perception: (1). in most cases distance between two atoms that is supposed to be connected is longer than the upper limit defined in our algorithm, and in some cases bonds were assigned to double by the length rules because of the short distance due to bad coordinates; (2). for some special structures, the rules in our algorithm cannot be applied correctly, and four structures are shown in Figure 3 as examples. As shown in Figure 3, the double bonds colored in red were predicted to be single by our algorithm. For these four structures the bond types of them are just beyond our rules. For example, for structure (a) in Figure 3, the length of the falsely predicted bond is 1.33 Å, suggesting that this bond has chance to be double by the length rule. While applying the conjugation rule, we found that cos of this bond is 0.60, which is larger than 0.50. Therefore this bond was set to single regardless of its bond length; however, it should be a double bond because it is in an aromatic ring. Thus, we can understand why the incorrect bond lengths in these structures cannot be handled by our algorithm because they are beyond the conventional values defined in our algorithm. We then used Discovery Studio [16], MOE [19] and SYBYL [17] to perceive the bond types for these 25 molecules that cannot be correctly predicted by our algorithm. We found that only one of them could be fixed by Discovery Studio and MOE, and none of the 19 molecules that have unusual bond lengths could be fixed. However, it is interesting to find that SYBYL can fix seven of them. That is to say, for these 25 molecules that cannot be correctly predicted by our algorithm, SYBYL performs better than Discovery Studio, MOE and even our algorithm.

Then, these 1290 molecules were minimized using the MMFF94 force field implemented in SYBYL [17]. Each structure in the sdf file was converted to the PDB format. After applying our algorithm, all molecules could be fixed correctly, suggesting that when the coordinates of the studied small molecules are reasonable the prediction accuracy of our algorithm can almost achieve 100%.

## Conclusions

We have presented a rule-based algorithm for automatic bond type perception in molecules given 3D atomic coordinates and element identities. Our algorithm is efficient to detect the structures when their geometries are reasonable. For many structures with coordinate errors, we can also perceive them by applying the small structural patterns.

However, there still remain some challenges, especially for molecules with misconnected and false connected bonds. Due to the absence of hydrogen in many structures we cannot determine whether bond efficiency is satisfied or not. And for the false connected bonds, we check the maximum connection number first, and detect the valence at last. In our algorithm we prefer to use small structural patterns to perceive structures instead of using a lot of large ones, and this will reduce the possibilities of perceiving structures incorrectly caused by similar structures. When fixing the isomers, we use the entropy of bond to select the best isomer.

In the near future we plan to improve our algorithm in two directions:

1. Connection detection: We plan to design and employ better rules to detect the bonds which should be connected but are ignored by our algorithm and to reduce the redundant bonds that are over-connected.

2. Define the entropy of bond by using more subtle scheme: To achieve this, a training process based on a lot of data is necessary. Some parameters and formula should be changed according to the training results.

## Methods

### Identification of connected atoms

First, connection between atoms should be judged according to Equation 1.

(1) $$0.8<d_{\text{ij}}<r_{i}+r_{j}+0.4$$

where *r*_*i*_ and *r*_*j*_ are the covalent radii of atoms *i* and *j* (the detailed data is listed in Additional file 1: Table S1[20]), and *d*_*ij*_ is the distance between them.

The right side of Equation 1 is derived by Meng and Lewis [11], and the left side was derived from our structural analysis based on 126,292 small molecules extracted from the ZINC database [21], and these molecules belong to the “all-purchasable” subset in ZINC. When the cutoff of 0.8 was not used and only the right side of Equation 1 was used to detect the connection of small molecules, 78 molecules had false connections. After adding the cutoff of 0.8, the number of the molecules with false predictions was reduced to 58. Therefore, in order to achieve more accurate predictions, the cutoff of 0.8 was employed. When bond *b*_*ij*_ was established, its initial bond order *O*_*ij*_ was set to 1.

This algorithm only judges the bond type between H, C, N, O, F, P, S, Cl, Br, and I, and the other kinds of bond order were simply set to 1. The bonds that have already been fixed would not be detected in the following steps, except that it is in a group and the group has many isomers.

### Over-connected correction

Due to the intrinsic property of each element, it should have the maximum connections. Here, we check whether C(4), N(4), P(4) or S(4) is over-connected (the number in parenthesis represents the maximum number of connections); if so, the longest bond is removed and the atom is check again until the number of the connections is no longer bigger than its maximum connection number. Here we only consider the above four kinds of elements, and the connections for the other elements are handled at the bottom of this algorithm.

### Hard rules

We employed some basic chemical rules to assign the bond types.

(1) We use *A*_*i*_ to denote atom *i*, and set its bonded number, *C*_*i*_, be the number of the atoms that are connected with this atom. If the number equals to

1.1 If the atom is hydrogen or halogen, *O*_*ij*_ is set to 1.

1.2 If the atom is sulfur and it connects to phosphorus, or the atom is nitrogen and it connects to sulfur, *O*_*ij*_ is set to 2.

1.3 If else, leave the bond unfixed and leave this rule.

(2) when *C*_*i*_ is 2 1 we apply the following rules:

2.1 If the atom is oxygen or sulfur, *O*_*ij*_ is set to 1 as the valence of oxygen and sulfur is 2.

2.2 *C*_*j*_ = max {*C*_*j*_, *C*_*k*_}, *C*_*k*_ = max {*C*_*j*_, *C*_*k*_}, if *Cj* = 1, skip step 2. 

2.3 Calculate the angle of two connected bonds (*b*_*ij*_ and *b*_*ik*_) using the following two equations:

(2) $$\theta ={\text{cos}}^{-1}\frac{\left(x_{i}-x_{j}\right)\cdot \left(x_{i}-x_{k}\right)}{\left|x_{i}-x_{j}||x_{i}-x_{k}\right|}$$

(3) $$\theta^{'}=\theta \cdot 180^{\circ }/\pi$$

where *x*_*i*_, *x*_*j*_ and *x*_*k*_ are the coordinates of atoms *i*, *j*, and *k*.

If *θ*^’^ is between 175° and 185°, these two bonds are considered to be linearly connected, and there are three kinds of possible schemes: when *C*_*j*_ equals to 2, suggesting that the bonded number of this atom is 2 and if the bond angle between its two bonds is between 175° and 185°, three adjacent bonds are therefore just in line. Here, we assume no continuous triple bond systems, and considering *b*_*ij*_ is in the middle of them, *O*_*ij*_ is set to 3 and then *O*_*ik*_ is set to 1. If *A*_*k*_ is C or N, *O*_*ij*_ is set to 1 and then *O*_*ik*_ is set to 2. In the other cases, both of *O*_*ij*_ and *O*_*ik*_ are set to 2. All models are applied if the previous ones do not work. 

(3) when *C*_*i*_ equals to 3

3.1 When the atom is N or P, and if it connects to oxygen (*A*_*j*_) and *C*_*j*_ = 1, this group is fixed using an acid model. If not, and all bond orders are set to be single.

3.2 When the atom is S, Cl, Br or I, and if it connects to oxygen (*A*_*j*_) and *C*_*j*_ = 1, this group is fixed as an acid. If not, leave it. 

**Figure 4:**
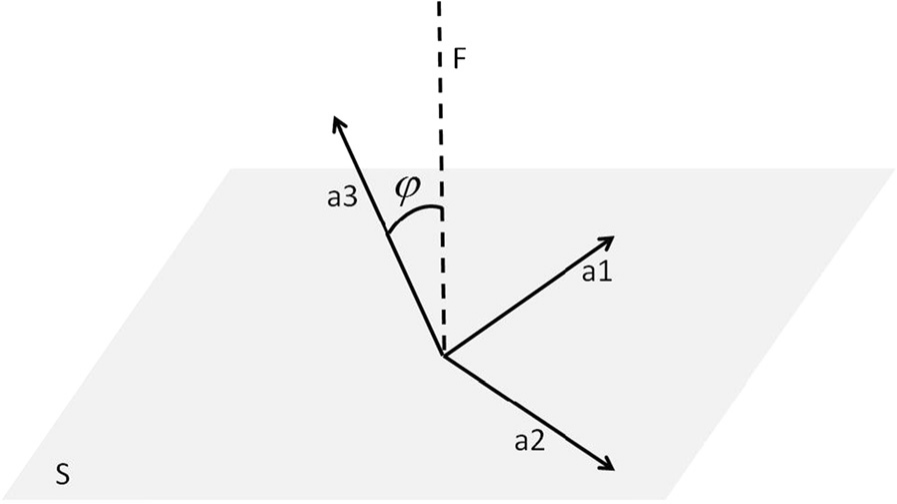
**The definition of ***φ*. **S is the plane that line a1 and line a2 forms**, **line F is the normal line of plane S**, **and ***φ ***is the angle between line F and line a3.**

3.3 When the atom is C, this atom and its connected atoms are put into one group, and the cos  of this group (Figure 4) is calculated using the following two equations:

(4) $$\begin{array}{l} \cos\varphi_{i}=\frac{\left(x_{i}-1-x_{i}\right)\times \left(x_{i}+1-x_{i}\right)\cdot \left(x_{i}+2-x_{i}\right)}{\left|\left(x_{i}-1-x_{i}\right)\times \left(x_{i}+1-x_{i}\right)||x_{i}+2-x_{i}\right|}i\\ \;=i\;\text{mod}\;k \end{array}$$

(5) $$\cos\varphi =\max\left\{\cos\varphi_{i}|i=\text{1…}k\right\}$$

**Figure 5:**
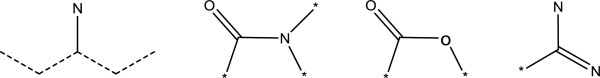
Patterns of atom C with the connection number of 3 (the dashed bond means that its order is unsure).

If the cos *ϕ* > 0.25, all the bonds connected by this atom are set to be single; otherwise, we will apply four models shown in Figure 5.

(4) When *C*_*i*_ equals to 4

4.1 If the atom is C or N, because the number of the maximum connections is 4, and when they are connected with 4 atoms, all the bonds should be single.

4.2 Else, if the atom is P, S, Cl or Br, I, it must be an acid group, then fix it.

### Length rules

For each bond *B*_*ij*_ connected to *A*_*i*_, the bond order is determined by the bond length. We use *Z*_*i*_ to denote the atomic number of *A*_*i*_, *Z*_*x*_ = min {*Z*_*i*_, *Z*_*j*_} and *Z*_*y*_ = max {*Z*_*i*_, *Z*_*j*_}.

1. If *A*_*x*_ is hydrogen, considering its valence, the bond order is set to 1; and if *A*_*y*_ is halogen, the bond order is also set to 1 because the acid model has already been considered in the hard rules.

2. The length of *B*_*ij*_ is defined as *L*_*ij*_. If this bond can be found in Table 3 (bond lengths in this table were obtained by a parameterization process to achieve the best prediction accuracy), the bond order is assigned by the following steps. It should be noted that the minor changes of the bond lengths listed in Table 3 do not change the prediction accuracy obviously, and the low sensitivity of bond lengths is also an advantage of our algorithm.

**Table 3:** **The reference bond lengths** (**Å**)

**Bond**	**L1**^***a***^	**L2**^***b***^
C-C	1.49	1.33
C-N	1.43	1.29
C-O	1.41	1.28
C-S	1.80	1.72
N-N	1.38	1.29
N-O	1.39	1.24

^*a*^L1 represents the length of single bond; ^*b*^L2 represents the length of double bond.

2.1 *L*_*ij*_ < *L*_2_: the bond length is smaller than the length of double bond, and the bond order is set to 2.

2.2 *L*_*ij*_ < *L*_2_ + 0.05: the bond type is set to 5, suggesting it is not sure, and it has better chance to be 2

2.3 *L*_*ij*_ < *L*_1_ − 0.04: the bond type is set to 4, suggesting that it is not sure.

2.4 *L*_*ij*_ < *L*_1_: the bond type is set to 6, suggesting that it has better chance to be 1.

2.5 For the rest unsigned bonds, their bond orders are set to 1.

3. If the bond cannot be found in Table 3, the bond type is set to 4.

Each step is applied after the application of the previous steps.

### Conjugation rules

After applying the previous steps, part of the bonds have been fixed, but there are still many unassigned bonds (bond type equals to 4, 5 or 6). Most of them have improper bond’s length and have the biggest chance to be aromatic. At this step, we design the conjugation rules to determine these unassigned bonds.

1. For bond *B*_*i*,*i* + 1_, atoms that connect to this bond, and *A*_*i*_, *A*_*i*_ + 1 are put into one group *G*(*k*)

If cos > 0.50, suggesting that they are totally not on the same plane, the bond order is then set to 1. If *O*_*i*, *i* + 1_=6, *C*_*i*_ ≥ 2, *C*_*i* + 1_ ≥ 2, *C*_*i*_ + 1≥2 and cos  > 0.2, they are likely not on the same plane, and the bond type of 6 indicates that the bond order has the chance to be single (*Oi*, *i* + 1=1).

2. The remaining unfixed connected bonds are divided into groups, and therefore we may have several groups for a molecule.

2.1 Group size equals to 1: We will check if this bond is connected with any double bond. If the bond is not connected with any double bond, and this bond and its neighbor bonds are on plane (when cos *ϕ* < 0.2, see above), the bond order is set to 2. There is one exception: if the bond is at the edge of the structure and the number of the bonds to which it are connected equals to 1, they are always on the same plane. In this condition, we will check if the bond type is 5 and the angle between these two bonds differs from 120°in 3 degrees. If not, the bond order is set to 1. When they are not on the same plane and the bond type is 5, we denote *BA*_*ij*_ as the bond angle between bond *i* and bond *j*

(6) $$BA=\min\left\{|BA_{\mathrm{ij}}-120|,j=\text{1…}n\right\}$$

where $\begin{array}{l} O12>4\\ O23=4 \end{array}$ equals to the number of the bonds that are connected to bond *i*. If *A*_2_ is smaller than 3, the bond order is then set to 2; and otherwise, the bond order is set to 1.

2.2 Group size equals to 2: we will get a return value after judging the previous bond orders that is larger than 3 and their neighbors of the two bonds (Table 4).

**Table 4:** **Return numbers based on small structural patterns**^***a***^

**Return number**	**0**	**1**	**2**	**4**	**5**
*Aii* doesn't have a double bond					
Bond types are 5 and 6					
*A*1 has more than 1 bonds					
*A*3 has more than 1 bonds					
$\begin{array}{l} O12=4\\ O23>4 \end{array}$					
$\begin{array}{l} O12>4\\ O23=4 \end{array}$					
*A*2 has three bonds					
Others			others		

^*a*^The dashed bond means this bond has already been fixed. The bold atom/bond means atoms/bonds around it are on the same plane. Conditions are taken into account from up to down. The plain bonds represents bonds to be fixed. Some fixed bonds are not given in the structures.

If return = 0, 1, 2: *O*_12_ =1 + return/2, *O*_23_ = 1 + return%2

If return = 4: *O*_12_ = 1, assign the other bonds using rule 2.1

If return =5: *O*_23_ = 1, assign the other bonds using rule 2.1

2.3 Fix isomers: For each bond, if one of the atoms is nitrogen and this nitrogen is only connected with one bond, the order is set to 1. For the others, a new rule is applied.

Rule: for one bond, if it connects to a fixed double bond, its order is set to 1; otherwise, we will check if one of its atoms just has three bonds and two of the bonds are single. If so, we will have the following two possibilities: (1). the bond type is 5, and the bond order is then set to 2; (2). the minimal angle between this bond and the connected bonds is larger or smaller than 120°in 3 degrees, the bond order is set to 1. If the above possibilities are not satisfied, the bond remains unfixed.

If there still remain bonds to be fixed after applying the above rule, for those unfixed ones, the order of one bond is set to 2 each time, and then the rule is applied again; if those bonds cannot be fixed, we will set the next bond and try the rule again. Once it has been done, we can get one isomer. Then, the bond order for the next unfixed bond is set to 2, and we will check if we can get another suitable structure. Thus, it is quite possible that we can get several isomers.

To determine the best structure from multiple isomers, we propose a new parameter: Entropy of bonds. For each isomer, the entropy of bonds is the number of bonds that satisfy the following criteria for each *B*_*ij*_:

1. *O*_*ij*_ = 1

2. it connected to at least two double bonds

3. The double bonds it connected at least connected to another bond.

Then the structure with the largest entropy is chosen as the best choice. If some isomers have the same entropy values, the structure that we first meet is chosen. Our results show that for most cases the representative structures can be chosen accurately using the easy definition.

### Fixing disordered bonds

We already checked the atoms to make sure they are not over-connected, but after the application of the rules above, many single bonds have been changed to double or triple bonds, and then some atoms are over-connected. Therefore, we use the maximum valence to judge if this atom is over-connected. For atom *A*_*i*_, the number of the connected bonds is calculated based on the following equation:

(7) $$iCon=\sum_{k=0}^{n}O_{\text{ik}}$$

If *iCon* > valence, the longest single bond is deleted, and the structure is checked again until *iCon* is no longer larger than the maximum valence. Here we just handle single bond, because for the bond with the bond order larger than 1, its bond length is reliable if this bond can be judged by the rules applied above.

## Competing interests

The authors declare that they have no competing interests.

## Authors' contributions

QZ wrote the paper, implemented the algorithm and ran tests; TH contributed to the paper and provided guidance; WZ, YL, JW, LZ ran tests; all authors read and approved the final manuscript.

## Supplementary Material

Additional file 1 **Table S1.** The radius of different elements; **Table S2.** The PDB codes for the structures used in the second data set. Click here for file

## References

[B1] Berman H Henrick K Nakamura H Markley JL The worldwide Protein Data Bank (wwPDB): ensuring a single, uniform archive of PDB data Nucleic Acids Res 2007 35 D301 D303 10.1093/nar/gkl971 17142228 PMC1669775

[B2] Spek AL Structure validation in chemical crystallography Acta Crystallogr D 2009 65 148 155 10.1107/S090744490804362X 19171970 PMC2631630

[B3] Yang LW Eyal E Chennubhotla C Jee JG Gronenborn AM Bahar I Insights into equilibrium dynamics of proteins from comparison of NMR and X-ray data with computational predictions Structure 2007 15 741 749 10.1016/j.str.2007.04.014 17562320 PMC2760440

[B4] Berman HM Westbrook J Feng Z Gilliland G Bhat TN Weissig H Shindyalov IN Bourne PE The protein data bank Nucleic Acids Res 2000 28 235 242 10.1093/nar/28.1.235 10592235 PMC102472

[B5] Dehof AK Rurainski A Bui QBA Bocker S Lenhof HP Hildebrandt A Automated bond order assignment as an optimization problem Bioinformatics 2011 27 619 625 10.1093/bioinformatics/btq718 21245051

[B6] Baber JC Hodgkin EE Automatic assignment of chemical connectivity to organic molecules in the Cambridge structural database J Chem Inf Comput Sci 1992 32 401 406 10.1021/ci00009a001

[B7] Bruno IJ Shields GP Taylor R Deducing chemical structure from crystallographically determined atomic coordinates Acta Crystallogr B 2011 67 333 349 10.1107/S0108768111024608 21775812 PMC3143025

[B8] Froeyen M Herdewijn P Correct bond order assignment in a molecular framework using integer linear programming with application to molecules where only non-hydrogen atom coordinates are available J Chem Inf Comput Model 2005 45 1267 1274 10.1021/ci049645z 16180903

[B9] Hendlich M Rippmann F Barnickel G BALI: automatic assignment of bond and atom types for protein ligands in the brookhaven protein databank J Chem Inf Comput Sci 1997 37 774 778 10.1021/ci9603487

[B10] Labute P On the perception of molecules from 3D atomic coordinates J Chem Inf Comput Model 2005 45 215 221 10.1021/ci049915d 15807481

[B11] Meng EC Lewis RA Determination of molecular topology and atomic hybridization states from heavy atom coordinates J Comput Chem 1991 12 891 898 10.1002/jcc.540120716

[B12] Sayle R PDB: Cruft to content (Perception of molecular connectivity from 3D coordinates) Daylight user meeting MUG01 2001

[B13] Zhao Y Cheng T Wang R Automatic perception of organic molecules based on essential structural information J Chem Inf Comput Model 2007 47 1379 1385 10.1021/ci700028w 17530839

[B14] Wang JM Wang W Kollman PA Case DA Automatic atom type and bond type perception in molecular mechanical calculations J Mol Graph Model 2006 25 247 260 10.1016/j.jmgm.2005.12.005 16458552

[B15] Ricketts EM Bradshaw J Hann M Hayes F Tanna N Ricketts DM Comparison of conformations of small molecule structures from the protein data bank with those generated by Concord, Cobra, ChemDBS-3D, and converter and those extracted from the Cambridge structural database J Chem Inf Comput Sci 1993 33 905 925 10.1021/ci00016a013

[B16] Discovery Studio 2.5 Guide, Accelrys Inc., San Diego 2009 http://www.accelrys.com

[B17] SYBYL-X 1.3 molecular simulation package 2011 St. Louis http://www.sybyl.com

[B18] Hou TJ Xia K Zhang W Xu XJ ADME evaluation in drug discovery. 4. Prediction of aqueous solubility based on atom contribution approach J Chem Inf Comput Sci2004a 44 266 275 10.1021/ci034184n 14741036

[B19] MOE, Chemical Computing Group Inc 2011 Montreal, Canada http://www.chemcomp.com/. 2011

[B20] Allen FH The Cambridge structural database: a quarter of a million crystal structures and rising Acta Crystallogr B 2002 58 380 388 10.1107/S0108768102003890 12037359

[B21] Irwin JJ Shoichet BK ZINC-a free database of commercially available compounds for virtual screening J Chem Inf Comput Model 2005 45 177 182 10.1021/ci049714+ PMC1360656 15667143

