# Supplementary material for: A rule-based algorithm for automatic bond type perception
Source: J Cheminform. 2012 Oct 31;4:26. doi: 10.1186/1758-2946-4-26 (PMC3557220; doi:10.1186/1758-2946-4-26)
Supplement: Additional file 1 — Table S1. The radius of different elements; Table S2. The PDB codes for the structures used in the second data set. [file 1758-2946-4-26-S1.doc]

**Supporting Materials**

Table S1. The radius of different elements (Å)

| Element | radius | Element | radius | Element | radius | Element | radius |
| --- | --- | --- | --- | --- | --- | --- | --- |
| H | 0.23 | Ar | 1.57 | Br | 1.21 | I | 1.40 |
| He | 0.93 | K | 1.33 | Kr | 1.91 | Xe | 1.98 |
| Li | 0.68 | Ca | 0.99 | Rb | 1.47 | Cs | 1.67 |
| Be | 0.35 | Sc | 1.44 | Sr | 1.12 | Ba | 1.34 |
| B | 0.83 | Ti | 1.47 | Y | 1.78 | La | 1.87 |
| C | 0.68 | V | 1.33 | Zr | 1.56 | Ce | 1.83 |
| N | 0.68 | Cr | 1.35 | Nb | 1.48 | Pr | 1.82 |
| O | 0.68 | Mn | 1.35 | Mo | 1.47 | Nd | 1.81 |
| F | 0.64 | Fe | 1.34 | Tc | 1.35 | Pm | 1.80 |
| Ne | 1.12 | Co | 1.33 | Ru | 1.40 | Sm | 1.80 |
| Na | 0.97 | Ni | 1.50 | Rh | 1.45 | Eu | 1.99 |
| Mg | 1.10 | Cu | 1.52 | Pd | 1.50 | Gd | 1.79 |
| Al | 1.35 | Zn | 1.45 | Ag | 1.59 | Tb | 1.76 |
| Si | 1.20 | Ga | 1.22 | Cd | 1.69 | Dy | 1.75 |
| P | 1.05 | Ge | 1.17 | In | 1.63 | Ho | 1.74 |
| S | 1.02 | As | 1.21 | Sn | 1.46 | Er | 1.73 |
| Cl | 0.99 | Se | 1.22 | Te | 1.47 | Tm | 1.72 |

Table S2. The PDB codes for the structures used in the second data set

| 1aaq | 1abe | 1abf | 1adb | 1add | 1adf | 1apb | 1apt | 1apu | 1apv | 1apw | 1aqb |
| --- | --- | --- | --- | --- | --- | --- | --- | --- | --- | --- | --- |
| 1bap | 1bra | 1bzm | 1cbx | 1cla | 1cps | 1csc | 1ctt | 1dbb | 1dbj | 1dbk | 1dbm |
| 1dhf | 1dih | 1dr1 | 1drf | 1dwb | 1dwc | 1dwd | 1ebg | 1ela | 1elc | 1etr | 1ets |
| 1ett | 1fbc | 1fbf | 1fbp | 1fkb | 1fkf | 1g6n | 1hbv | 1hpv | 1hsl | 1htf | 1htg |
| 1hvi | 1hvj | 1hvk | 1hvr | 1hvs | 1l83 | 1ldm | 1lgr | 1lyb | 1mbi | 1mcb | 1mcf |
| 1mch | 1mcj | 1mcs | 1mdq | 1mfe | 1mnc | 1nnb | 1pgp | 1phe | 1phf | 1phg | 1phh |
| 1ppc | 1pph | 1ppk | 1ppl | 1ppm | 1pso | 1rbp | 1rne | 1rnt | 1rus | 1snc | 1sre |
| 1tha | 1tlp | 1tmn | 1tmt | 1tng | 1tnh | 1tni | 1tnj | 1tnk | 1tnl | 1ulb | 1xli |
| 2ak3 | 2cgr | 2csc | 2ctc | 2dbl | 2dri | 2er6 | 2gbp | 2ifb | 2ldb | 2mcp | 2phh |
| 2pk4 | 2r04 | 2rnt | 2rtd | 2sns | 2tmn | 2xim | 2xis | 2ypi | 3cla | 3cpa | 3csc |
| 3dfr | 3fx2 | 3pgm | 3ptb | 3tmn | 3tpi | 4cla | 4dfr | 4fab | 4gr1 | 4hvp | 4mdh |
| 4phv | 4sga | 4tim | 4tln | 4tmn | 4ts1 | 4xia | 5abp | 5acn | 5cna | 5cpp | 5enl |
| 5hvp | 5icd | 5ldh | 5p21 | 5sga | 5tim | 5tln | 5tmn | 5xia | 6abp | 6apr | 6cpa |
| 6enl | 6gst | 6rnt | 6tim | 6tmn | 7abp | 7acn | 7cat | 7cpa | 7est | 7hvp | 7tim |
| 7tln | 8abp | 8atc | 8cpa | 8hvp | 8icd | 8xia | 9aat | 9abp | 9hvp | 9rub |  |
